# *Porphyromonas gingivalis* induced periodontitis exacerbates progression of non‐alcoholic steatohepatitis in rats

**DOI:** 10.1002/cre2.41

**Published:** 2016-09-28

**Authors:** Ryutaro Kuraji, Hiroshi Ito, Miyako Fujita, Hitomi Ishiguro, Shuichi Hashimoto, Yukihiro Numabe

**Affiliations:** ^1^ Department of Periodontology School of Life Dentistry at Tokyo, Nippon Dental University 1‐9‐20 Fujimi Chiyoda‐ku Tokyo 102‐8159 Japan; ^2^ Nippon Dental University 1‐9‐20 Fujimi Chiyoda‐ku Tokyo 102‐8159 Japan

**Keywords:** animal model, endotoxemia, high‐fat diet, non‐alcoholic fatty liver disease, periodontal bacteria

## Abstract

Non‐alcoholic steatohepatitis (NASH) is a chronic liver disease that can develop into hepatocirrhosis and hepatic carcinoma. In recent years, epidemiological and animal studies have reported that *Porphyromonas gingivalis (P. gingivalis)*, a known periodontopathic bacteria, is closely related to NASH. However, previous studies could not demonstrate a direct relationship between periodontitis, *P. gingivalis* infection, and NASH. The purpose of the present study was to examine the impact of *P. gingivalis*‐associated periodontitis on the onset and progression of NASH. Forty‐two male Wistar rats were used in this study. Rats were fed a high‐fat diet (HFD) for 12 weeks in order to induce fatty liver. At 4 weeks from the start of feeding, the animals were performed ligature placement around the maxillary first molar tooth in order to induce experimental periodontitis, and then a *P*. *gingivalis* slurry was applied around the ligature twice in a week for 8 weeks (HFD/Pg(+) group). Controls were given the slurry without *P. gingivalis* after ligature placement using the same protocol (HFD/Pg(−) group). Significant increases in alveolar bone resorption and inflammation in periodontal tissue around the molar tooth in the HFD/Pg(+) group were observed when compared with the HFD/Pg(−) group. Moreover, histological images showing NASH characterized by perivenular lipid deposition including big fatty drops, ballooning degeneration, and focal necrosis with inflammatory cells were confirmed in the liver of rats in the HFD/Pg(+) group. Significant increases in alanine aminotransaminase, aspartate aminotransferase, and C‐reactive protein levels were observed in the HFD/Pg(+) group. Furthermore, endotoxin levels in serum in the HFD/Pg(+) group were significantly higher than those in the HFD/Pg(−) group. The present study demonstrated that experimental periodontitis induced by *P. gingivalis* led to the progression of NASH in rats with fatty liver. Increased levels of endotoxin derived from *P. gingivalis* infection appear to play a considerable role in the progression of NASH.

## 1 INTRODUCTION

Non‐alcoholic fatty liver disease (NAFLD) is a disease entity that comprises a range of histopathological findings, including non‐alcoholic fatty liver (NAFL) with good prognosis and non‐alcoholic steatohepatitis (NASH) with poor prognosis (Ludwig, Viggiano, McGill, & Oh, 1980; Angulo, 2002). NASH is a phenotype of liver diseases in metabolic syndrome, and it has been reported that NASH is closely related to the severe lifestyle diseases represented by obesity and diabetes (Sanyal, 2011; Leite et al., 2011). Furthermore, it has been reported that NASH develops into liver diseases such as hepatocirrhosis and hepatic carcinoma (Liou & Kowdley, 2006; Abdelmalek & Diehl, 2007).

The two‐hit theory proposed by Day has been widely supported as explaining the mechanism of NASH onset and progression (Day & James, 1998; Day, 2002). Initially, a fatty liver is induced as “the first hit” by hepatic fat accumulation arising from lifestyle diseases. Then, “the second hit” by various factors related to liver injury induces hepatic inflammation and fibrosis in the fatty liver. In recent years, the effects of endotoxin derived from enterobacteria have been reported, and it is known that hyperreactivity against lipopolysaccharide is led in fatty liver in cases of obesity (Wigg, Roberts‐Thomson, Dymock, McCarthy, Grose, & Cummins, 2001; Farhadi et al., 2008).

*Porphyromonas gingivalis* (*P. gingivalis*) is a primary causative agent of periodontitis (Socransky, Haffajee, Cugini, Smith, & Kent, 1998; Hajishengallis, Darveau, & Curtis, 2012; Hajishengallis et al., 2011), and a number of studies have detected *P. gingivalis* in various organs throughout the body as well as in the oral cavity; thus, it might be a risk factor for systemic diseases including cardiovascular disease, diabetes, and rheumatoid arthritis (Seymour, Ford, Cullinan, Leishman, & Yamazak, 2007; Pizzo, Guiglia, Russo, & Campisi, 2011; Figuero & Sanchez‐Beltran, 2011). A recent epidemiological study showing that *P. gingivalis* at high frequency was present in NAFLD/NASH patients suggested a close relationship between periodontitis and NASH (Yoneda et al., 2012). Furusho et al. reported acceleration of hepatic inflammation and fibrosis as a result *of P. gingivalis* infection in the dental pulp of a maxillary molar in mice with fatty liver fed with high‐fat diet (HFD; Furusho et al., 2013).

It is therefore possible that *P. gingivalis* infection acts as “the second hit” for progression into NASH. However, direct administration via the vein or dental pulp is a major method in previous animal studies on NASH. Periodontal tissue is covered by gingival epithelium, which acts as a physical and biological barrier (Tonetti, Imboden, Gerber, Lang, Laissue, & Mueller, 1994; Dale et al., 2001). It has not been verified whether *P. gingivalis* infection via periodontal tissue is associated with NASH. Here, we investigated the progression of NASH caused by *P. gingivalis*‐induced experimental periodontitis as the second hit in a rat fatty liver model.

## 2 MATERIAL AND METHODS

### 2.1 Animals and experimental groups

All experimental procedures were approved by the animal care committee of the School of Life Dentistry at Tokyo, Nippon Dental University.

**Figure 1:**
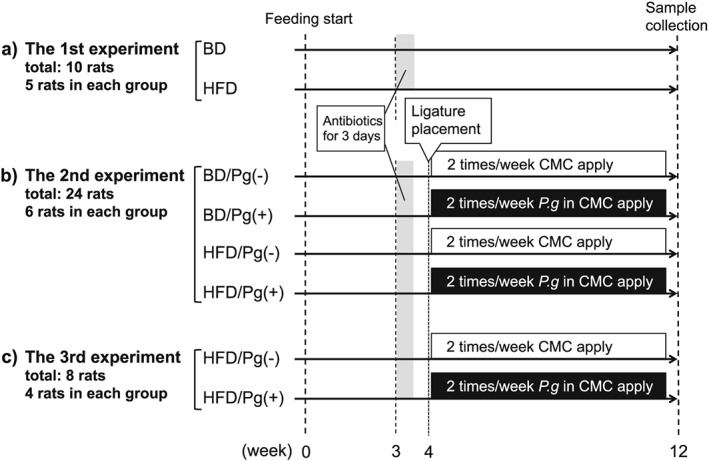
Study design. BD, basal diet; HFD, high‐fat diet; *P.g*, *Porphyromonas gingivalis;* CMC, sodium carboxymethyl cellulose

Forty‐two 8‐week‐old male Wistar rats (Clea Japan, Osaka, Japan) were used for three experiments in this study as follows (Figure 1).

1. The first experiment (*n* = 10): This experiment was carried out to establish the fatty liver model. Five rats were fed either an HFD (60% fat, Research Diets No. D12492; Research Diets, New Brunswick, NJ) or a basal diet (BD; 10% fat, Research Diets No. D12450B) for 12 weeks. These feeding groups were named as the HFD and BD groups, respectively. No other interventions were included in this experiment.

2. The second experiment (*n* = 24): This experiment was carried out to assess the combined effect of HFD feeding, ligature placement, and *P. gingivalis* oral infection on a rat maxilla and liver. After receiving ligatures around the right maxillary first molar of the rats fed with BD or HFD the animals were divided into four groups of six animals each, as follows: BD/Pg(−), BD/Pg(+), HFD/Pg(−), and HFD/Pg(+). Animals pertaining to the Pg(+) group were infected with *P. gingivalis* as described below.

3. The third experiment (*n* = 8): This experiment was carried out to evaluate endotoxin and C‐reactive protein (CRP) levels in serum. Animals were divided into two groups of four animals each, as follows: HFD/Pg(−) and HFD/Pg(+).

All rats were raised in cages at 23°C and 50% humidity under a 12‐hr light–dark cycle. After 12 weeks, rats were fasted for 16 hr, and blood was collected via the carotid artery under general anesthesia with intraperitoneal injection of sodium pentobarbital (50 mg/kg). They were then sacrificed, and the right maxilla and caudate lobe of the liver were extracted.

### 2.2 Ligation around cervical region of maxilla molar

At a week before treatment of ligature placement, all rats used in this experiment were given 1 mg/ml sulfamethoxazole and 200 μg/ml trimethoprim in drinking water for 3 days to reduce the native oral flora (Nakada, Kato, & Numabe, 2015).

Because it has become known from the process of the first experiment that fatty liver was led by HFD feeding for 4 weeks, experimental periodontitis was performed at 4 weeks from the start of feeding. The circumference of the cervical region of the right maxillary first molar (Ml) was ligated with 3‐0 silk sutures in a state of open mouth using Hashimoto's gag (Nonaka Rikaki, Tokyo, Japan) under general anesthesia. The knot of suture was fixed with composite resin to the mesial site of M1.

### 2.3 Preparation and application of *P. gingivalis* slurry

*Porphyromonas gingivalis* strain W83 was grown at 37°C for 7 days on agar plates containing brain heart infusion (Becton Dickinson, New Jersey) supplemented with 10% defibrinated horse blood, 5 mg/ml hemin, and 0.5 μg/ml menadione under anaerobic conditions (AnearoPack system; Mitsubishi Gas Chemical, Tokyo). Colonies were then selected and anaerobically subcultured at 37°C for 48 hr in brain heart infusion broth supplemented with hemin and menadione. *P. gingivalis* turbidity (OD 0.8) was spectrophotometrically determined at 600 nm, and bacteria were mixed with 4% sodium carboxymethyl cellulose (Wako Pure Chemical Industries, Tokyo; Jain, Batista, Serhan, Stahl, & Van Dyke, 2003).

For 8 weeks from to sacrifice, 100 μl of bacterial slurry was applied around the ligature of the MI twice in a week. Rats treated with or without *P. gingivalis* were named as the Pg(+) group or Pg(−) group, respectively.

### 2.4 Microfocus X‐ray computed tomography (μCT) analysis of maxilla

The extracted right maxilla was fixed in 3% paraformaldehyde solution for 48 hr, and the following analysis was performed.

The maxilla was scanned using a μCT system (ELESCAN II; Nittetsu Elex, Tokyo). The μCT scanning was performed on the horizontal plane parallel to the molar occlusal plane of the maxilla, the sagittal plane parallel to the dentition of molar teeth, and the coronal plane parallel to the tooth axis. Measurement conditions for μCT were as follows: tube voltage, 80 kV; electrical current, 44 mA; image pixel size, 512 × 512; and slice thickness, 30 μm. After scanning, the degree of alveolar bone resorption was evaluated in accordance with the method of Tokunaga et al. (2011) on three‐dimensional images produced using computer software (TRI/3D‐BON Version 7.0; Ratoc System Engineering, Tokyo). In three regions (mesial, central, and distal sites) of the Ml, the distance from the palatal cement‐enamel junction (CEJ) to the alveolar bone crest (ABC) was measured, and the average of three regions was defined as a representative value for each rat.

### 2.5 Histopathological analysis of maxilla

After μCT scanning, the maxilla was decalcified in 10% ethylenediaminetetra‐acetic acid for 4 weeks and then embedded in paraffin. Sagittal sections (6 μm) parallel to the plane including the distal root of Ml and the mesial root of the second molar were prepared from the palatal view using a microtome (RM2145; Leica, Hessen, Germany). Samples were stained with hematoxylin and eosin (H‐E), and histological observation was performed using a light microscope (Microphot‐FX; Nikon, Tokyo).

### 2.6 Analysis of biochemical parameters and endotoxin in blood

Serum was obtained from the blood by centrifugation at 10,000 × g for 60 min and was stored at −80°C. Activities of aspartate aminotransferase (AST) and alanine aminotransaminase (ALT) were analyzed using the JSCC transferable method (L‐type wako GOT‐J2/L‐type wako GPT‐J2; Wako) in order to assess liver function. CRP was measured with a rat CRP ELISA kit (Immunology Consultants Laboratory, Portland, OR). Endotoxin was quantified with Endospecy ES‐50M Set (Seikagaku Corporation, Tokyo).

### 2.7 Histopathological analysis of liver tissue

The caudate lobe of the liver was fixed in 3% paraformaldehyde solution for 24 hr and then embedded in paraffin or frozen. Paraffin sections (5 μm) were stained with H‐E and Azan–Mallory (Muto Pure Chemicals, Tokyo), and 8‐μm frozen sections prepared with a cryostat (CM3050S; Leica, Hessen, Germany) were stained with Sudan III (Muto). Three different fields (100× magnification, 346 × 260 μm) with a focus on the central vein were randomly selected on Sudan‐stained images, and orange‐stained liver lipid deposition was measured using ImageJ analysis software (Schneider, Rasband, & Eliceiri, 2012).

Furthermore, NAFLD activity score (NAS) of the liver was evaluated in accordance with the definition of Kleiner et al. on H‐E and Sudan staining image to diagnose liver tissue (Kleiner et al., 2005). Total NAS in individual rats was calculated as the sum of three scores of steatosis, inflammation, and cell injury (ballooning) and is shown in terms of means and standard deviation of intra‐group scores (*n* = 6).

### 2.8 Detection of *P. gingivalis* in subgingival plaque, blood, and liver

A subgingival plaque sample was collected at the start of the experimental period and at sacrifice in order to examine the presence of *P. gingivalis*. Three sterile paper points were inserted into the pockets of distal‐palatal sites in Ml for 10 s and were stored in 0.3 ml of phosphate‐buffered saline.

The DNA in subgingival plaque, serum, and liver was extracted from each sample using QIAamp DNA Mini Kits (Qiagen, Hilden, Germany). *P. gingivalis* and total bacteria were then quantified by real‐time polymerase chain reaction (PCR) using Taqman MGB probes and primers (Thermo Fisher Scientific, Waltham, MA; Table 1.) corresponding to 16S ribosomal RNA. Amplification was carried out in Taqman® Fast Advanced Master Mix (Thermo) containing extracted DNA, probes, and primers using a StepOnePlus™ real‐time PCR system (Thermo) under the following conditions: 20 min at 95°C, followed by 40 cycles per 1 min at 95°C, and 20 min at 60°C. Data were analyzed using StepOne^1 M^ software version 2.1 (Thermo). Tenfold serial dilutions of DNA of known concentration were used to construct standard curves for quantification of *P. gingivalis* and total bacteria. The bacterial detection threshold is 100 cells, and coefficients of variation among the Cycle threshold values for *P. gingivalis* and total bacteria averaged 1.4% (ranged from 0.9 to 2.0%) and 1.4% (ranged from 0.6 to 3.2%), respectively.

**Table 1:** Taqman probe and primer

Target	Sequence (5′‐3′)
*Porphyromonas gingivalis*	
Forward primer	CTTGACTTCAGTGGCGGCAG
Reverse primer	TCAGTCGCAGTATGGCAAGCT
Probe	TGAAATGCATAGATATCACG
Universal (total bacteria)	
Forward primer	TGCGGGACTTAACCCAACA
Reverse primer	TGGAGCATGTGGTTTAATTCGA
Probe	CACGAGCTGACGACARC*

* "R" indicates mixed sequence of A and G bases.

### 2.9 Statistical analysis

The power analysis was performed based on data from a pilot study, relative to the mean difference and SD between four intragroups (by the second experiment protocol) for the distance from CEJ to ABC and serum ALT levels using G*Power 3 analysis software (Faul, Erdfelder, Lang, & Buchner, 2007 May ). Effect sizes for two variables were 1.05 and 1.24, respectively. The sample size was calculated assuming normal distribution to obtain power value of 0.8 with α error 0.05, and minimum four rats in each group were needed using one‐way analysis of variance (ANOVA).

Normal distribution patterns for respective data were demonstrated by Kolmogorov–Smirnov test. ANOVA and Tukey test were then used to compare four groups, and Student's *t*‐test was used for comparisons between two groups. Further data analysis on NAFLD activity score was performed by Kruskal–Wallis *H*‐test and Mann–Whitney *U*‐test with Bonferroni correction as nonparametric data. All calculations were performed using statistical software (SPSS ver.15.0 j; IBM, Chicago, IL). A *P* value of <0.05 was considered to be significant, and results are shown as means ± SD.

## 3 RESULTS

### 3.1 The first experiment: Preparation of fatty liver by feeding with HFD

After 12 weeks, rats in the HFD group showed higher body weight when compared to those in the BD group (499 ± 22 vs. 451 ± 25 g, *P* < 0.05; *n* = 5).

**Figure 2:**
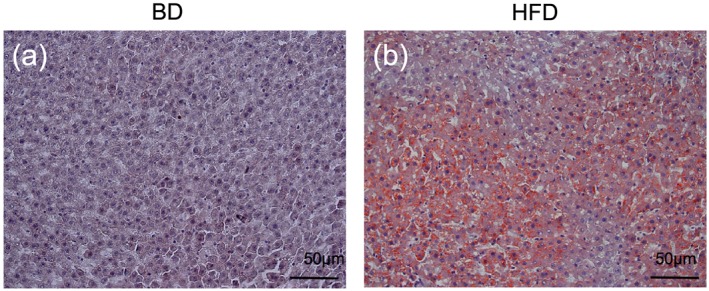
Fatty liver after high‐fat diet (HFD) feeding. In order to evaluate the effects of HFD feeding on the liver, rats fed HFD (60% fat, 20% carbohydrate, and 20% protein) were classed as the (a) HFD group. Rats fed basal diet (BD) (10% fat, 70% carbohydrate, and 20% protein) were defined as the (b) BD group (control). Sudan III‐stained images of the hepatic lobulus were also observed

Serum ALT levels in the HFD group were significantly higher than those of the BD group (42.6 ± 4.8 vs. 29.6 ± 5.8 g, *P* < 0.05; *n* = 5). However, AST levels did not differ between the two groups (82.4 ± 8.7 vs. 77.4 ± 8.7 g, *P* = 0.39; *n* = 5). Lipid deposition in hepatocytes in the HFD group was markedly observed on Sudan‐stained images (Figure 2a and b). These results revealed that HFD feeding induced mild fatty liver in rats.

### 3.2 The second experiment: Effect of experimental periodontitis induced with ligature placement and *P. gingivalis* on fatty liver rats

#### 3.2.1 Alveolar bone loss and periodontitis after HFD feeding and *P. gingivalis*‐infection

After rats fed with BD or HFD were treated with or without *P. gingivalis* around the ligature of the first molar twice a week, the degree of periodontitis in the BD/Pg(−), BD/Pg(+), HFD/Pg(−), and HFD/Pg(+) groups was determined.

**Figure 3:**
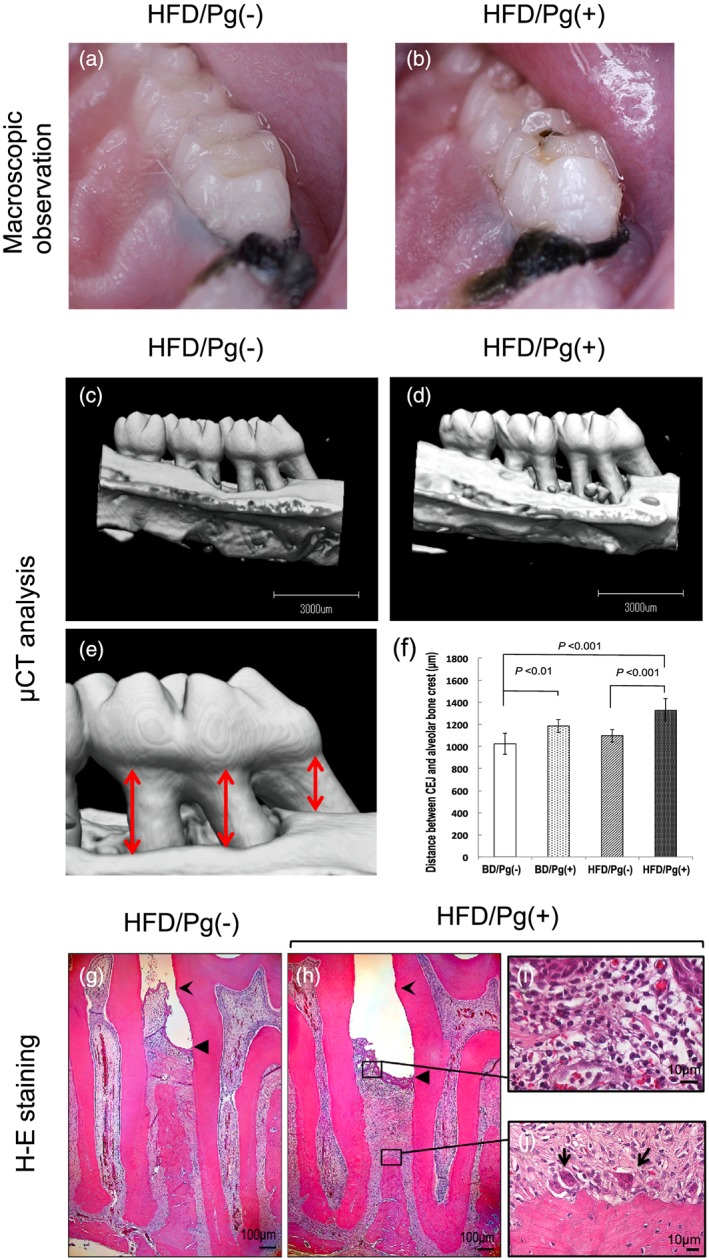
Experimental periodontitis induced by ligature placement and *P Porphyromonas gingivalis* infection. Rats fed high‐fat diet (HFD) for 4 weeks were treated around the ligature of the first molar with or without *P. gingivalis*. After 12 weeks, photographs of the right maxillary molars were taken of (a) Pg(−) group and (b) Pg(+) group), and microfocus X‐ray computed tomography (μCT) analysis was performed on (c) Pg(−) group and (d) Pg(+) group. Subsequently, the average distance from cement‐enamel junction (CEJ) to alveolar bone crest in (e) three palatal regions (mesial, central, and distal sites) of right maxillary first molar M1 was measured on (f) three‐dimensional images produced by μCT imaging. Data are shown as means ± SD (*n* = 6). The results of observation on hematoxylin and eosin (H‐E) staining images showed that progression of attachment loss, infiltration of inflammatory cells, and disorder of collagen fibers in the (h) Pg(+) group were more severe when compared with those in the (g) Pg(−) group (forked arrowhead, CEJ; triangle arrowhead, epithelial junction). Inflammatory cells mainly composed of (i) lymphocytes and (j) the appearance of osteoclasts on the alveolar bone crest (round‐tipped arrow) were confirmed when magnifying the black frame in the (h) Pg(+) group

Intraoral images shown in Figure 3a and b are representative observations in rats of the HFD group at 8 weeks after *P. gingivalis* application. More severe redness and swelling at the marginal gingiva in the Pg(+) group were observed as compared with that in the Pg(−) group.

Results for the distance from CEJ to ABC on the μCT images are shown in Figure 3c–f. *P. gingivalis* infection markedly exacerbated the destruction of alveolar bone in both the BD and HFD groups. Alveolar bone loss in the BD/Pg(+) group increased 15.7% (*P* < 0.01; *n* = 6) in comparison to that in the BD/Pg(−) group, and the alveolar bone loss in the HFD/Pg(+) group increased by 20.9% (*P* < 0.001) in comparison to that in the HFD/Pg(−) group. Furthermore, bone loss in the HFD/Pg(+) group was 12.0% (*P* < 0.05), significantly higher than that in the BD/Pg(+) group (Figure 3d).

The effects of *P. gingivalis* application on the ligated periodontal tissue were observed by histological analysis. Distance from the CEJ to the junctional epithelium in the Pg(+) group was greater than that in the Pg(−) group, and the attachment loss in the Pg(+) group showed greater progression in the apical direction along the cementum (Figure 3g and h). Hypertrophy of the epithelium, severe infiltration by inflammatory cells, disorder of collagen fibers, and alveolar bone resorption by osteoclasts were also observed in the Pg(+) group. However, similar findings were not observed in the Pg(−) group (Figure 3i and j).

#### 3.2.2 Levels of bacteria in dental plaque, blood, and liver after HFD feeding and *P. gingivalis* infection

In order to assess the internal presence of *P. gingivalis* passing through the periodontal tissue, DNA was extracted from dental plaque, blood, and liver of treated rats (*n* = 6) and was analyzed by real‐time PCR. The results are shown as a percentage of *P. gingivalis* among total bacteria. *P. gingivalis* was not detected in the plaque before the bacterial infection but was present at 17.0 ± 13.3% in the plaque at 8 weeks after infection. However, *P. gingivalis* was not detected in the serum or liver (detection limit: 5 cells/μl of serum, 40 cells/g of liver). These results suggested that *P. gingivalis* colonizes local tissue at the site of periodontitis and did not diffuse widely in this study.

#### 3.2.3 Liver function after HFD feeding and *P. gingivalis* infection

**Figure 4:**
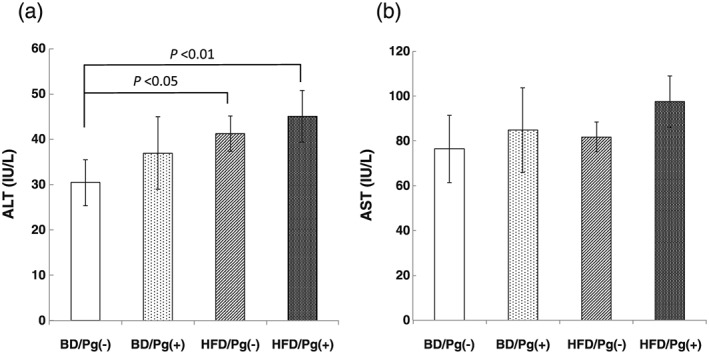
Serum levels of liver function parameters. Serum (a) alanine aminotransaminase (ALT) and (b) aspartate aminotransferase (AST) were measured using the enzymatic method, after 12 weeks of feeding with basal diet (BD) or high‐fat diet (HFD). Data are shown as means ± SD (*n* = 6)

Serum ALT and AST activities were analyzed at 8 weeks after treatment of ligature placement and *P. gingivalis*. ALT activities in the HFD/Pg(−) and HFD/Pg(+) groups (*n* = 6) were significantly higher than those in the BD/Pg(−) groups (Figure 4a). ALT in the BD/Pg(+) and HFD/Pg(+) groups was also higher than those in the BD/Pg(−) and HFD/Pg(−) groups, respectively, but the increase in ALT after bacterial infection was not significant. On the other hand, in AST activities between groups, there was no significant difference by ANOVA (Figure 4b). However, AST in the HFD/Pg(+) group was 1.2‐fold higher than that in the HFD/Pg(−) group when using Student's *t*‐test.

#### 3.2.4 Liver histology after HFD feeding and *P. gingivalis* infection

**Figure 5:**
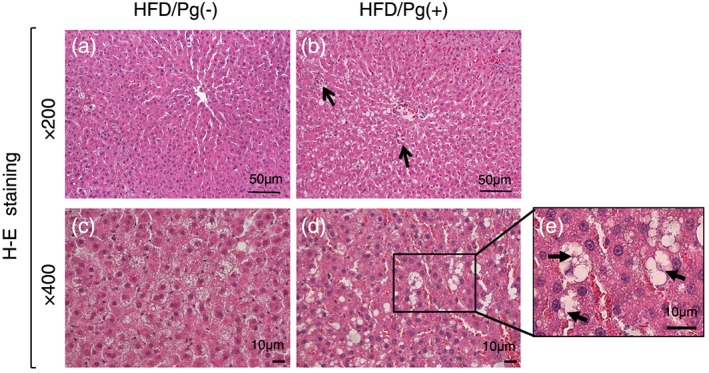
Hematoxylin and eosin (H‐E) stained images of liver. The caudate lobes of liver were extracted from rats in the high‐fat diet (HFD)/Pg(−) group, (a) ×200 and (c) ×400, and the HFD/Pg(+) group, (b) ×200 and (d) ×400 (arrow: focal necrosis), for diagnosis of NASH, and fields with a focus on the central vein were observed in H‐E stained images. Large fatty drops and focal hepatocyte necrosis (b, arrow) were confirmed in the HFD/Pg(+) group, and ballooning degeneration with (e) Mallory−Denk bodies (arrow) was observed on magnified images.

Perivenous inflammation and lipid deposition were observed on H‐E stained liver sections, thus suggesting NASH. Slight perivenous lipid deposition, small fatty drops, and a few infiltrating neutrophils were observed in the HFD/Pg(−) group (Figure 5a).

In contrast, moderate lipid deposition, numerous large fat drops, and some focal necrosis with neutrophilic infiltration were observed around the central vein in the HFD/Pg(+) group (Figure 5b). Furthermore, numerous ballooning degenerations with Mallory–Denk bodies were confirmed on high magnification (×400) images in the HFD/Pg(+) group (Figure 5c–e). These observations in the HFD/Pg(+) group suggested that the liver of rats treated with HFD feed and *P. gingivalis* infection progressed to NASH.

**Figure 6:**
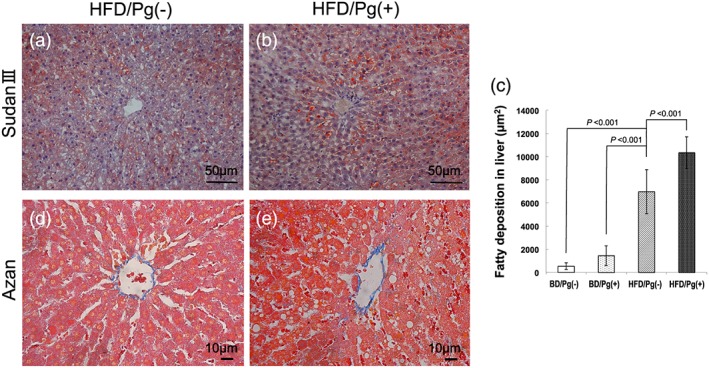
Lipid and fibrosis stained images in the liver after high‐fat diet (HFD) feeding and *Porphyromonas gingivalis* infection. The lipid staining images with Sudan III on (a) Pg(−) group and (b) Pg(+) group were observed to specifically evaluate lipid deposition and large fatty drops after 12 weeks of feeding with HFD. The orange‐stained area was quantitatively measured using ImageJ in (c) three randomly selected fields. Data are shown as means ± SD (*n* = 6). In addition, Azan–Mallory stained images of (d) Pg(−) group and (e) Pg(+) group were observed to evaluate hepatic fibrosis

Sudan III staining was performed on frozen liver sections in order to analyze the degree of lipid deposition and fatty drops. Greater perivenular fatty drop formation was observed in the HFD/Pg(+) group when compared with the HFD/Pg(−) group (Figure 6a and b). Furthermore, it was confirmed on the ImageJ analysis that *P. gingivalis* infection increased liver lipid deposition by 48% (*P* < 0.001; *n* = 6) in the HFD/Pg(−) group (Figure 6c).

Furthermore, Azan–Mallory staining was performed to estimate hepatic fibrosis. However, perivenous and pericellular fibroses were not confirmed in the sections of either the HFD/Pg(−) or HFD/Pg(+) group (Figure 6d and e).

The frequency distribution of NAS in the respective groups showed that HFD feeding and *P. gingivalis* infection accelerated progression to NASH from fatty liver (Table 2). Total NAS in the HFD/Pg(+) group was significantly higher than that in the HFD/Pg(−) group (4.83 ± 1.47 vs. 2.17 ± 0.75, *P* < 0.01; *n* = 6). Total NAS of 5 or more in rats was only seen in the HFD/Pg(+) group in this study. A total NAS of 5 more or over in human cases is considered to indicate NASH.

**Table 2:** NAFLD activity score in rat liver

Histological finding	(Score)	BD/Pg(−) (*n* = 6)	BD/Pg(+) (*n* = 6)	HFD/Pg(−) (*n* = 6)	HFD/Pg(+) (*n* = 6)
Steatosis (%)					
<5	(0)	6	3	0	0
5–33	(1)	0	3	6	3
33–66	(2)	0	0	0	3
>66	(3)	0	0	0	0
Lobular Inflammation					
NO foci	(0)	6	1	1	0
<2 foci per 200 field	(1)	0	4	5	3
2–4 foci per 200 field	(2)	0	1	0	2
>4 foci per 200 field	(3)	0	0	0	1
Cell injury—ballooning					
None	(0)	6	5	4	0
Few balloon cells	(1)	0	1	2	4
>4 balloon cells	(2)	0	0	0	2

NAFLD, non‐alcoholic fatty liver disease. NAFLD activity score was scored according to the criteria of Kleiner et al. (2005).

#### 3.2.5 The third experiment: Serum levels of endotoxin and CRP in fatty liver rats treated with ligature placement and *P. gingivalis*

**Figure 7:**
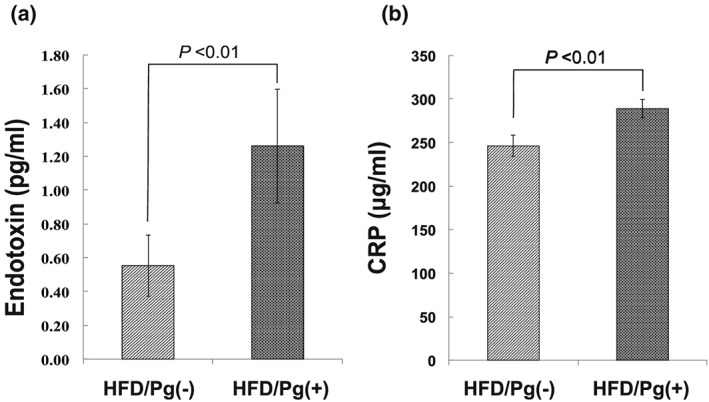
Serum levels of endotoxin and C‐reactive protein (CRP) after high‐fat diet (HFD) feeding and *Porphyromonas gingivalis* infection. (a) Serum endotoxin and (b) CRP levels were respectively measured using ELISA and enzyme colorimetry after 12 weeks of feeding with HFD. Data are shown as means ± SD (*n* = 4).

Serum endotoxin levels in the HFD/Pg(+) group were 2.4‐fold higher (*P* < 0.01, n = 4) than those in the HFD/Pg(−) group (Figure 7a).

Levels of CRP, which is synthesized in hepatocytes activated by upregulation of tumor necrosis factor‐α and interleukin 6, in the HFD/Pg(+) group increased by 17% (*P* < 0.01) when compared with those the HFD/Pg(‐) group (Fig. 7B).

## 4 DISCUSSION

It has been reported that various factors are associated with the pathogenesis of NASH, including oxidative stress, insulin resistance, and adipocytokines (Sanyal, 2011; Malaguarnera, Rosa, Nicoletti, & Malaguarnera, 2009). However, it is unclear whether other factors are related to the onset and progression of NASH. Therefore, elucidating the mechanisms for the pathogenesis of NASH may be valuable for drafting prophylactic and therapeutic strategies. *P. gingivalis* is part of a bacterial group called the keystone, of which a small amount plays a central role in the progression of chronic periodontitis (Hajishengallis et al., 2012; Hajishengallis et al., 2011). Lipopolysaccharide and various proteases produced by *P. gingivalis* are known to be associated with various systemic diseases, in addition to periodontal pathogens (Seymour et al., 2007; Mikuls, 2009). Yoneda *et al*. reported that the detection frequency of *P. gingivalis* in saliva from NASH patients was significantly higher than that in healthy subjects and that infection by *P. gingivalis* may be an additional risk factor for NASH (Yoneda et al., 2012).

Previous animal experiments showed that intravenous injection (Yoneda et al., 2012) and direct application via dental pulp (Furusho et al., 2013) of *P. gingivalis* exacerbated steatohepatitis in murine models fed HFD. However, the results in these experiments were not related to periodontitis induced by *P. gingivalis* infection.

The gingival epithelium in healthy periodontal tissue covers connective tissue, including blood vessels, and ordinarily obstructs the biofilm adhering on the root surface (Tonetti et al., 1994; Dale et al., 2001). In diseased tissues, increases in permeability and microulceration of the gingival epithelium allow invasion of microbial products, but the covering of epithelium is maintained continuously or intermittently (Müller‐Glauser & Schroeder, 1982; Hujoel, White, García, & Listgarten, 2001). Various reports (Forner, Larsen, Kilian, & Holmstrup, 2006; Lockhart et al., 2008) have suggested that *P. gingivalis* itself does not readily invade in the blood circulation via healthy periodontal tissue. Therefore, in this study, *P. gingivalis* infection was induced in experimental periodontitis after ligating the cervical region of the maxillary molar tooth (Ml) but was not detected in the blood and liver in the HFD/Pg(+) group by real‐time PCR.

Ligature placement around the cervical region of molars is the conventional method for inducing experimental periodontitis in rats (Oz & Puleo, 2011). There is clear evidence demonstrating more severe horizontal bone loss in murine models infected with *P. gingivalis* and other Gram‐negative bacteria in addition to this ligature placement (Samejima, Ebisu, & Okada, 1990; Amar, Zhou, Shaik‐Dasthagirisaheb, & Leeman, 2007). The combination of ligature placement and *P. gingivalis* infection also accelerated the destruction of periodontal tissue and bone resorption as compared to ligature placement without *P. gingivalis* in this study.

When rats were fed HFD for 12 weeks, increased body weight and development of mild fatty liver were observed in the animals (Figure 2), similarly to a previous report (Anstee & Goldin, 2006).

Progression into NASH induced by *P. gingivalis* infection was confirmed in HFD groups (Figures 4−6) but was not seen in BD groups (data not shown). Our results show that fatty liver induced by both HFD and *P. gingivalis* infection through periodontitis increases the risk of NASH progression.

Yoneda et al. reported that the direct injection of *P. gingivalis* into the jugular vein in mice fed HFD increased ALT levels in the blood and lipid deposition in the fatty liver (Yoneda et al., 2012). Furthermore, Furusho et al. reported that when *P. gingivalis* was administered into the pulp chamber in steatosis model mice fed HFD, hepatic lipid deposition and focal fibrosis developed in mice with periapical lesions, as compared to control animals without *P. gingivalis* infection (Furusho et al., 2013). These observations largely agree with our results using experimental periodontitis. However, hepatic fibrosis was not confirmed in our animal model. Fibrosis is not necessary for a diagnosis of NASH, but it is one of the factors that determine the stage of NASH. We believe that these differences are the result of different *P. gingivalis* administrative methods.

On the other hand, it has been reported that oral gavage administration of *P. gingivalis* in the BD‐fed mice increased hepatic fat and triglyceride levels (Arimatsu et al., 2014). However, in the present study, when *P. gingivalis* was pasted to the periodontal tissue of the rats fed HFD without ligature placement, periodontitis in the maxilla was not developed, and ALT and AST levels did not increase in the blood (data not shown). Furthermore, lipid deposition and inflammatory cells were scarcely observed in the liver. These results suggest that both periodontitis and *P. gingivalis* infection are important factors in the progression of NASH.

The present observation that *P. gingivalis* was not confirmed in the blood and liver reminded us of other pathogens: proinflammatory cytokines, proteases, and endotoxin. Endotoxemia derived from enterobacteria is related to the pathogenesis of NASH (Wigg et al., 2001; Farhadi et al., 2008). Moreover, Imajo et al. reported that upregulation of CD14 by leptin‐STAT3 led hyperreactivity against low‐dose lipopolysaccharide in fatty liver induced by HFD (Imajo et al., 2012). It was reported that endotoxemia is derived from experimental gingivitis in human subjects after cessation of all oral hygiene measures (Wahaidi, Kowolik, Eckert, & Galli, 2011), as well as from enterobacteria. In this study, *P. gingivalis* infection induced increases in serum endotoxin and CRP levels (Figure 7).

One limitation of our study is that while the ligature alone model is basically an inflammatory model, to add *P.gingivalis* or any other bacteria in this model increases the complexity. Namely, the results observed might be due to total burden of disease in the complex model and not due to *P. gingivalis* itself. Further studies are required to elucidate the several mechanisms involved in the relationship between periodontitis, *P. gingivalis* and NASH.

In conclusion, the present study demonstrated that *P. gingivalis* infection via experimental periodontitis in rats fed HFD induced NASH. Furthermore, we believe that *P. gingivalis* and its endotoxin play a critical role in the relationship between periodontitis and NASH.
